# The impact of gender on long-term mortality in patients with multivessel disease after primary percutaneous coronary intervention

**DOI:** 10.1007/s12471-015-0754-x

**Published:** 2015-10-08

**Authors:** S. Ghauharali-Imami, M. Bax, A. Haasdijk, C. Schotborgh, P. Oemrawsingh, J. Bech, R. van Domburg, F. Zijlstra

**Affiliations:** 1 Haga-Teaching Hospital, HagaZiekenhuis, Leyweg 275, 2545 CH The Hague, The Netherlands; 2 Medical Centre Haaglanden, The Hague, The Netherlands; 3 Reinier de Graaf Gasthuis, Delft, The Netherlands; 4 Erasmus MC, Rotterdam, The Netherlands

**Keywords:** ST-elevation myocardial infarction, Gender, Complete revascularisation, Mortality

## Abstract

**Background:**

Gender and complete revascularisation are known to affect mortality. The objective of this study was to analyse a gender difference in mortality with respect to complete revascularisation for multivessel disease after primary percutaneous coronary intervention (PPCI) in ST-elevation myocardial infarction (STEMI) patients.

**Method:**

In a prospective consecutive cohort of 1472 patients presenting with STEMI for PPCI, between January 2006 and January 2010, 832 patients with multivessel disease were analysed. The primary outcome was long-term mortality.

**Results:**

Median follow-up was 3.3 ± 1.2 years. Complete revascularisation was performed less in females than in males (30 vs. 38 %; *p* = 0.04). At PPCI, women (27 %, *n* = 221) were ± 10 years older (*p* = 0.001), had more hypertension, renal failure and symptoms of heart failure (all *p* < 0.01). Cumulative long-term mortality with incomplete revascularisation was higher in females (F: 30 vs. M: 15 %, *p* = 0.01). After adjustment for baseline characteristics, complete revascularisation (0.84; 95 % CI 0.54–1.32) and gender (1.11; 95 % CI 0.73–1.69) lost significance. Also the gender-by-complete revascularisation interaction was not significant at long term. In women, age under 60 years independently predicted higher mortality (HR 10.09; 95 % CI 3.08–33.08; *p* < 0.001).

**Conclusion:**

In STEMI patients with multivessel disease at PPCI, women under the age of 60 years had higher mortality, but in women older than 60 years comorbidity impacted the outcome of revascularisation strategy in the long term.

## Introduction

Gender and revascularisation strategy (complete vs. incomplete revascularisation) are factors known to affect mortality in patients with ST-elevation myocardial infarction (STEMI) and multivessel disease [1–3]. Evidence of a higher mortality in females dates back to the fibrinolysis era [4], particularly in patients presenting with STEMI (20 vs. 8 %, *p* = 0.029) [4, 5]. Compared with fibrinolysis, a primary percutaneous coronary intervention (PPCI) has been proven to be a better reperfusion strategy [6], especially for women [7–9]. Some researchers ascribe the higher mortality in women presenting with STEMI to higher age and more comorbidity at presentation or to less effective treatment and fewer drug-eluting stents [7, 10]. Others found gender to be an independent predictor for mortality [11].

Currently, the optimal revascularisation strategy for multivessel disease is a matter of debate [3, 12] and the impact of gender has not been investigated yet. Therefore, the objective of this study was to investigate long-term mortality in relation to complete revascularisation for multivessel disease in males and females with PPCI for STEMI. Furthermore, possible interactions for gender-by-treatment strategy and gender-by-age for mortality were analysed in this patient group.

## Methods

**Fig. 1:**
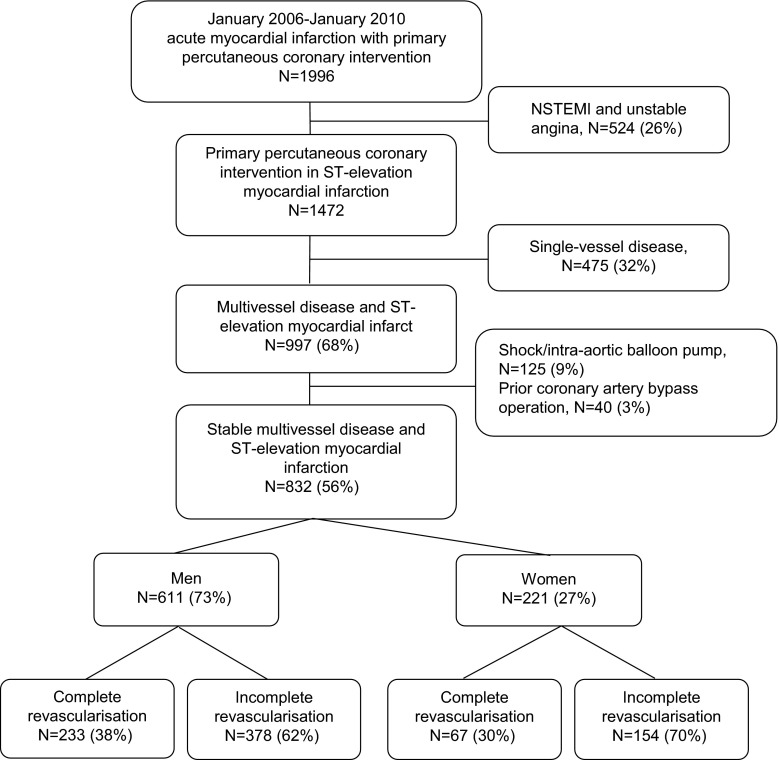
Flowchart of patient inclusion and revascularisation strategy for multivessel disease according to gender after primary percutaneous coronary intervention for ST-elevation myocardial infarction

From January 2006 to January 2010, 1472 consecutive patients were prospectively registered with PPCI for STEMI [13, 14], of whom 997 (50 %) had multivessel disease. According to local protocol, all patients with multivessel disease, except for patients with multivessel PCI (7 %), were adjudicated for revascularisation strategy by the ‘heart team’ (93 %, at least one interventional cardiologist and one heart surgeon). Multivessel disease was defined as > 50 % diameter stenosis by visual estimate in one or more non-culprit epicardial coronary arteries or their major branches on PPCI angiography. Patients with isolated left main coronary stenosis were classified as having two-vessel disease. Patients with prior coronary artery bypass graft (CABG), cardiogenic shock, an intra-aortic balloon pump, mechanical ventilation or resuscitation at presentation for PPCI were excluded (*n* = 165; 17 %). As presented in Fig. 1, the remaining 832 (56 %) patients with multivessel disease were analysed according to gender and revascularisation strategy: incomplete revascularisation, in which only the culprit lesion was treated at PPCI versus complete revascularisation of all significant non-culprit lesions during the PPCI procedure or in a staged session (PCI: median; 18 days, IQR: 12–40 days or CABG: median; 16 days, IQR 8–46 days).

## Clinical variables

Hospital records and angiographic images were reviewed for procedural data and clinical variables including age, gender, smoking habit, use of medication or history of diabetes, hypertension, hypercholesterolaemia, previous STEMI, previous PCI or CABG, renal failure (glomerular filtration rate (GFR) < 30 ml/min) at the time of presentation for PPCI. During follow-up, data on repeat revascularisation (PCI or CABG), recurrent myocardial infarction, occurrence of cerebrovascular accidents and mortality were recorded. Mortality was verified at the Dutch national population registry (‘Statistics Netherlands’) in The Hague. Follow-up was achieved in hospital in 100 % and during follow-up in 99 %, as only 1 % of patients migrated.

## Medical treatment from ambulance to catheterisation laboratory

All patients presenting within 6 h from the onset of symptoms with STEMI received a loading dose of clopidogrel 600 mg orally, acetylsalicylic acid (250 mg intravenously or 300 mg orally) and 5000 IU heparin at first medical contact (either in the ambulance or at the emergency room). The use of glycoprotein IIb-IIIA inhibitors and/or low-molecular-weight heparin before, during or after PPCI was at the operator’s decision. A procedure was considered successful when < 20 % of residual stenosis was achieved by balloon angioplasty or stent deployment at the culprit lesion, with Thrombolysis in Myocardial Infarction (TIMI) grade 3 flow.

## Discharge medication

After PPCI, clopidogrel 75 mg/day was maintained for at least 12 months and acetylsalicylic acid 80 mg/day indefinitely. Further prescription was left to the treating cardiologist.

## Endpoints

The main purpose of this study was to investigate long-term mortality in relation to complete revascularisation for multivessel disease in males and females with PPCI for STEMI. Furthermore, possible interactions for gender-by-treatment strategy and gender-by-age for mortality were analysed in this patient group.

## Statistical analysis

Quantitative data are expressed as mean ± SD and nominal data as frequencies followed by percentages (*n*; %). Data were checked for a normal distribution and analysed by either the independent sample T-test or Chi-square test when applicable. Nominal data were analysed using a Chi-square test or a Mann-Whitney U test when appropriate. Data with a normal distribution are presented as mean ± standard deviation and skewed data as median with interquartile range (IQR) 25th−75th percentile. Data are censored at end of life or end of follow-up (September 2012). Crude mortality was analysed by the Kaplan-Meier method using the log-rank test. Logistic regression was used for the analysis of in-hospital mortality. Proportional Cox regression analysis was utilised for 1-year and long-term mortality in women versus men and was expressed as a hazard ratio with 95 % confidence interval followed by significance (HR; 95 % CI, *p*-value). All baseline characteristics were at first analysed in the univariate and secondly only the most significant characteristics were added to gender and treatment strategy in the multivariate analysis according to a maximum of 10 % of the number of in-hospital events (1 event; age), at 1-year (4 events; age, time to first medical contact, renal failure at admission (GFR < 0.30 ml/mmol), procedural success) and in the long-term (7 events; age, time to first medical contact, renal failure at admission (GFR < 0.30 ml/mmol), procedural success, previous acute myocardial infarction, positive family history, hypertension). Age was analysed according to categories of 5 years, starting from 40 years of age (less than 40 vs. more than 40 years, etc.) and stratified to gender.

All tests were two-sided, a *p*-value < 0.05 was considered statistically significant. Statistical analysis was performed using SPSS statistical software for Windows.

## Results

As presented in Table 1, women were significantly older (71.2 ± 12.1 vs. 61.8 ± 12.3, *p* < 0.001), more often had hypertension, symptoms of heart failure and renal failure at admission than men (all *p* < 0.01). Smoking, familial predisposition, and previous PCI were more frequent in men (all *p* < 0.05). Previous STEMI and median time from symptom to first medical contact was comparable between genders. Complete revascularisation for multivessel disease was more frequent in men than in women (38 vs. 30 %, *p* = 0.04). There was an equal distribution of two- and three-vessel disease, culprit and non-culprit lesions. During PPCI, glycoprotein IIb-IIIa inhibitors were more often used in males than in females (62 vs.72 %, *p* = 0.005). No significant gender difference was shown for TIMI flow pre- or post-PPCI, the number of stents placed and the stent length. However, the diameter of the stent was smaller in females than in males (3.01 vs. 3.15 mm, *p* = 0.002).

**Table 1:** Baseline and procedural characteristics of ST-elevation myocardial infarct patients with multi-vessel disease at primary percutaneous intervention

Baseline characteristics *N* (%)	Female (*n* = 221; 27 %)	Male (*n* = 611; 73 %)	*P*
Age (mean ± SD)	71.2 ± 12.1	61.8 ± 12.3	0.001
Diabetes mellitus	46 (21)	128 (21)	NS
Hypercholesterolaemia	54 (24)	126 (21)	NS
History renal failure	7 (3)	17 (3)	NS
Hypertension	111 (50)	223 (37)	< 0.001
Family history	66 (30)	233 (38)	0.03
Smoker	71 (32)	259 (42)	0.008
Symptoms of heart failure	15 (7)	16 (3)	0.005
Previous percutaneous coronary intervention	10 (5)	53 (9)	0.046
Previous acute myocardial infarction	17 (8)	47 (8)	NS
GFR < 0.30 ml/mmol at presentation	58 (29)	80 (14)	< 0.001
**Procedural characteristics** *n* **(%)**			
Median; 25th–75th percentile			
Symptom-first medical contact (hours)	1:42.(1:00–4:00)	1:33 (0:45–3:06)	0.08
First medical contact-needle (hours)	0:35.(0:10–1:13)	0:30 (0:10–1:15)	0.40
Needle-balloon (hours)	0:15 (0:11–0:21)	0:16 (0:11–0:22)	0.52
Incomplete revascularisation	154(70)	378 (62)	0.04
Complete revascularisation after PPCI	67 (30)	233 (38)	
1-stage PCI	11 (4)	50 (8)	NS
2-stage PCI	28 (13)	95 (16)	NS
CABG	28 (13)	88 (14)	NS
Three-vessel disease	114 (51)	294 (48)	NS
Culprit			
Left anterior descending	87 (39)	219 (36)	NS
Right coronary artery	100 (45)	262 (43)	NS
Left circumflex	32 (15)	123 (21)	NS
Left main	1 (1)	0 (0)	NS
Non-culprit			
Left anterior descending	109 (49)	329 (54)	NS
Right coronary artery	84 (38)	224 (37)	NS
Left circumflex	133(60)	343 (56)	NS
Left main	35 (6)	18 (8)	NS
Glycoprotein IIb/IIIa inhibitor	133 (62)	432 (72)	0.005
TIMI-flow pre-primary percutaneous intervention			
0	141 (64)	435 (71)	NS
1	33 (15)	68 (11)	
2	32 (15)	78 (13)	
3	15 (7)	30 (5)	
TIMI-flow post- primary percutaneous intervention			
0	5 (2)	6 (1)	NS
1	7 (3)	8 (1)	
2	17 (8)	53 (9)	
3	192 (87)	544 (89)	
Procedural success	185 (84)	521(85)	NS
Drug-eluting stent	86 (40)	196 (32)	NS
Bare metal stent	132 (60)	411 (68)	0.04
Total number of stents	1.45 ± 0.91	1.46 ± 0.83	NS
Diameter stents	3.01 ± 0.51	3.15 ± 0.52	0.002
Length of stent	19.9 ± 7.8	20.7 ± 8.3	NS

## Mortality

**Fig. 2:**
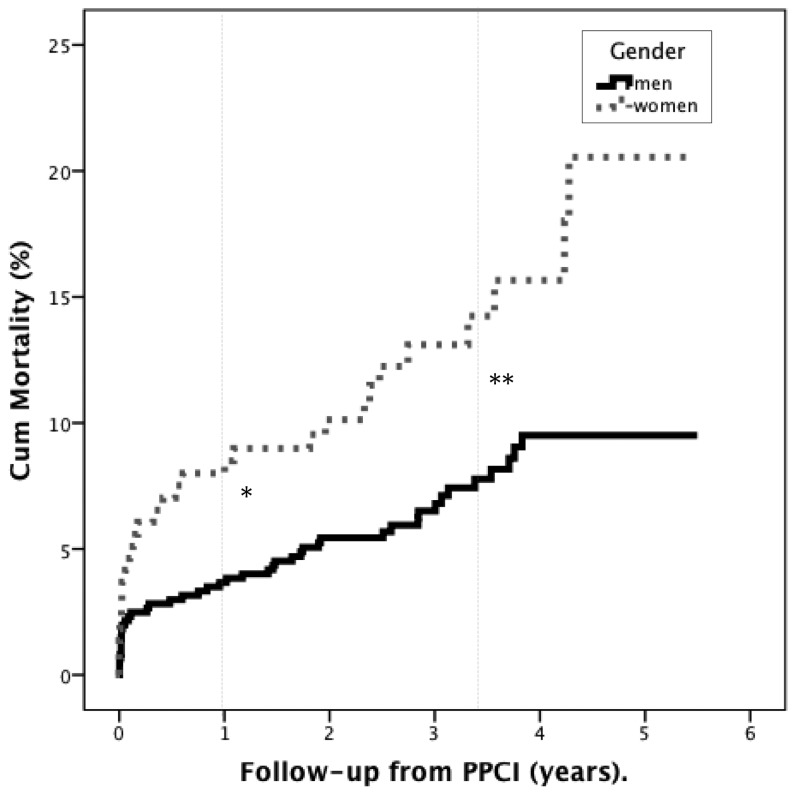
Long-term mortality in male and female patients with multivessel disease after primary PCI for ST-elevation myocardial infarction. A significantly higher mortality in women was shown at 1-year, *p = 0.009 and at long term, **p=0.004

As presented in Fig. 2, in-hospital mortality was similar in men and women (M: 2 vs. F: 3 %, *p* = 0.27). In the long-term, cumulative mortality was higher in women than in men (at 1 year: 8 vs. 4 %, *p* = 0.009 and at long-term: 13 vs. 7 %, *p* = 0.004). Overall, complete revascularisation resulted in lower cumulative mortality than incomplete revascularisation (in-hospital: 1 vs. 3 %, *p* = 0.009, at 1-year: 2 vs. 6 %, *p* = 0.007 and long term: 7 vs. 10 %, *p* = 0.08).

## Mortality in females vs. males according to treatment strategy

As shown in Table 2, in women no significant difference for mortality was shown between complete or incomplete revascularisation during follow-up, whereas in men a significantly lower mortality was shown for complete revascularisation in hospital (*p* < 0.001).

**Table 2:** Cumulative data from Kaplan-Meier analysis with *p*-values according to the log-rank test in females versus males for treatment strategy during follow-up

Cumulative mortality	Complete revascularisation (CR)			Incomplete revascularisation (ICR)			ICR vs. CR in females	ICR vs. CR in males
Gender	Female (%, number of events)	Males (%, number of events)	*P*	Females (%, number of events)	Males (%, number of events)	*P*	*P*	*P*
In-hospital	5 % (3)	0 % (1)	0.005	3 % (4)	3 % (10)	0.65	0.46	< 0.001
1-year	5 % (3)	1 % (3)	0.11	9 % (19)	5 % (14)	0.05	0.24	0.02
Long-term	9 % (6)	6 % (14)	0.32	15 % (23)	8 % (30)	0.01	0.23	0.30

## Mortality in females vs. males according to treatment strategy

**Fig. 3:**
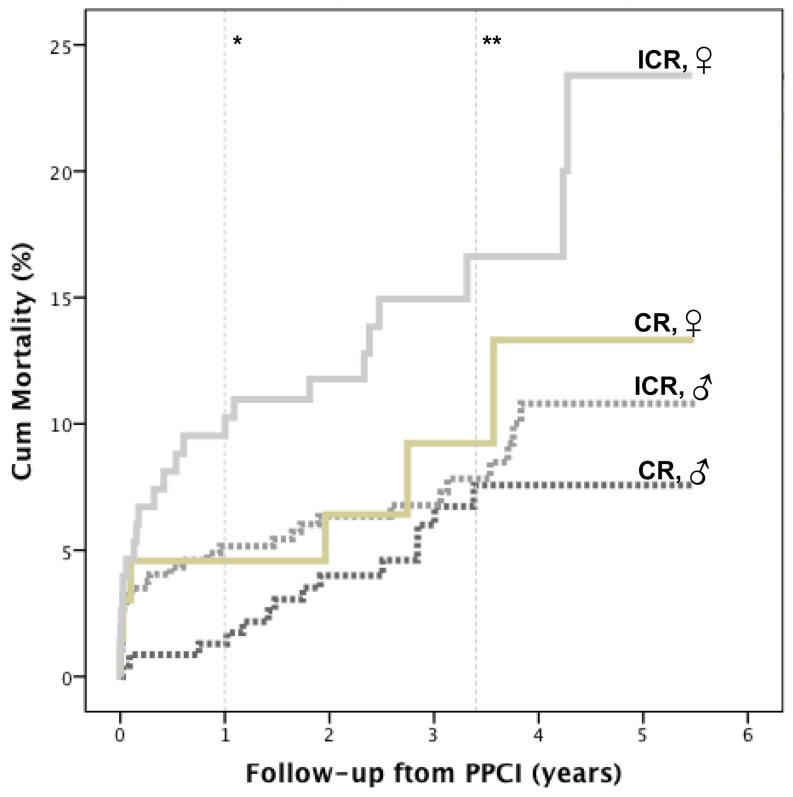
Mortality according to treatment strategy in females and males. Kaplan-Meier: in the long term women benefit more from complete than from incomplete revascularisation, at 1 year*, *p* = 0.05 and at long term **, *p* = 0.01; *CR* complete revascularisation*, ICR* incomplete revascularisation, *♂* male, *♀* female

As presented in Fig. 3, complete revascularisation had higher in-hospital mortality in females than in males (*p* = 0.005), but similar long-term mortality. Incomplete revascularisation had comparable in-hospital mortality between genders, but higher cumulative 1-year and long-term mortality in females.

## Mortality in females vs. males according to age

**Fig. 4:**
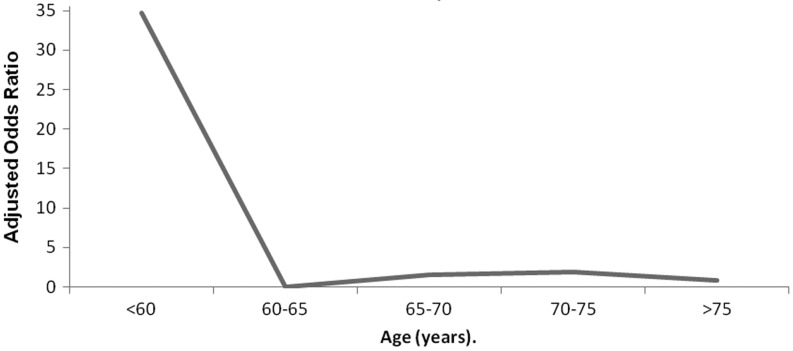
Age-adjusted long-term mortality in women compared with men at long-term follow-up. Females under the age of 60 years had a significantly higher mortality rate compared with males of a similar age

A significant gender disparity in age at presentation for PPCI was shown in our analysis (Table 1). As presented in Fig. 4, when mortality in men was compared with women of a similar age, higher mortality was shown in women under the age of 60 years.

## Univariate analysis

As presented in Table 3, complete revascularisation had lower mortality than incomplete revascularisation during follow-up, however not in hospital. Females had higher mortality than males at 1-year and also in the long term. The interaction of complete revascularisation-by-gender and age-by-gender was not significant (*p* = 0.73). The interaction of age 60 years-by-gender was significant at 1-year and in the long term only (*p* = 0.03 and *p* = 0.002, respectively).

**Table 3:** Univariate and multivariate analysis for mortality according to gender and treatment strategy adjusted for; in-hospital: age at primary percutaneous intervention, at 1-year adjusted also for time to first medical contact, renal failure at admission (GFR < 0.30 ml/mmol), TIMI-flow post procedure and at long-term also for previous acute myocardial infarction, positive family history, hypertension

	In-hospital				1-year				Long-term			
	Univariate HR; 95 %CI	*P*	Multivariate HR; 95 %CI	*P*	Univariate HR; 95 %CI	*P*	Multivariate HR; 95 %CI	*P*	Univariate HR; 95 %CI	*P*	Multivariate HR; 95 %CI	*P*
Female gender	1.78; 0.68–4.66	0.24	0.91; 0.33–2.54	0.86	2.30; 1.21–4.35	0.01	1.13; 0.52–2.43	0.76	2.29; 1.59–3.31	< 0.001	1.11; 0.73–1.69	0.63
Complete revascularisation	0.50; 0.16–1.53	0.23	0.62; 0.20–1.94	0.41	0.32; 0.14–0.77	0.01	0.36; 0.13–0.95	0.04	0.63; 0.42–0.94	0.02	0.84; 0.54–1.32	0.45
Age at primary PCI	1.08; 1.04–1.12	< 0.001	1.08; 1.03–1.12	0.001	1.08; 1.06–1.12	< 0.001	1.05; 1.02–1.09	0.002	1.08;1.07–1.10	< 0.001	1.07;1.05–1.09	< 0.001

## Multivariate analysis

As shown in Table 3, after adjustment for baseline characteristics gender no longer predicted mortality and complete revascularisation was only associated with 1-year morality. The interaction for age under 60 years-by-gender (*p* = 0.006) showed a higher mortality rate in females than in males under the age of 60 years in the long term, independent of risk profile (HR 10.09; 95 % CI 3.08–33.08, *p* < 0.001).

## Discussion

The main finding of our long-term analysis was that females benefit from complete revascularisation equally to males. In addition, incomplete revascularisation had higher mortality in females than in males in the long term. However, significance was lost after adjustment for baseline characteristics. In addition, the gender-by-complete revascularisation and gender-by-age interaction was not significant in the long term. However, when mortality was analysed between genders according to age, a significantly higher mortality was shown in females compared with males under the age of 60 years, independent of risk profile.

This is an important topic because recent literature has shown better clinical outcome with complete revascularisation in patients with multivessel disease [3]. This is also underlined by the Culprit study, with a lower rate of the composite endpoint in patients with complete revascularisation (HR: 0.45; 95 % CI: 0.24–0.84; *p* = 0.009) [15]; however, no significance was shown for mortality. Our analysis shows an overall lower mortality with complete revascularisation. However, complete revascularisation was performed significantly less often in women than in men, whereas no significant difference was shown for three-vessel disease, culprit or non-culprit lesions. In addition, incomplete revascularisation had higher long-term mortality in females than in males. Previous research has shown less aggressive management strategies with lower frequencies of PCI and less aggressive medical treatment in women [16, 17]. Women in our analysis had smaller vessels and more often renal failure at admission and this might have complicated a complete revascularisation strategy. According to Gevaert et al. [18] renal failure at admission is an independent predictor for in-hospital mortality, however with similar impact between genders.

The unadjusted mortality was higher in women during follow-up, but reduced significantly after adjustment for risk profile. This is in accordance with recent research [1, 19–21], and underlines the importance of aggressive management of comorbidity during follow-up. In a meta-analysis involving 18,555 women with STEMI and PPCI, Pancholy et al. found a nearly twofold higher risk for mortality in women in hospital, which was significantly attenuated when adjusted for baseline cardiovascular risk factors and clinical profile [22]. In contrast to Pancholy et al., in our analysis in-hospital mortality was similar between genders. This was also shown in other research [1, 10] and largely explained by equal symptom to first medical contact, first medical contact-to-needle and needle-to-balloon times in men and women in our cohort [23, 24]. Furthermore, the homogenous patient cohort, with the exclusion of cardiac shock patients and the prerequisite of a PPCI in order to enter our analysis, might also have reduced a gender difference for in-hospital mortality [25].

Further, a significantly higher age at admission in females was noticed in our analysis and also shown in previous studies. A protective effect of oestrogens has been suggested to explain the older age and higher risk profile at presentation for PPCI in females [26].

A comparison between mortality in men and women of similar age showed a higher mortality rate in females than in males under the age of 60 years, independent of risk profile. A higher mortality in younger women was also shown in previous studies [1, 27–29].

## Limitations

A possible selection bias was minimised in our study as the revascularisation strategy for non-culprit lesions was decided by the ‘heart team’ (including at least one cardiologist and one heart surgeon) in 93 % of patients and was not subject to an individual opinion. Furthermore, with the exclusion of cardiac shock and previous CABG, a homogenous cohort was analysed and these limits strengthen our database. Moreover, shock is inherently related to a higher mortality, especially in women with STEMI [23–25]. Also patients were followed-up for 3.3 ± 1.2 years, and this increases our power. We tried to adjust for important confounders with either logistic or Cox regression analysis. However, the number of events in our cohort limited us in the number of confounders we could adjust for. Although we assent to the importance of bleeding complications in females at PPCI and its possible influence on mortality, it was not included in our database. Medical treatment in hospital was according to protocol; however the medication at discharge was not listed in our database and might have influenced long-term mortality. In summary, our cohort represents contemporary clinical practice and although no definitive conclusions can be drawn, we were able to present equal benefit from complete revascularisation compared with higher mortality risk with incomplete revascularisation in women than in men in the long term. This study emphasises the need for a large prospective randomised trial with the power to investigate and specify optimal treatment strategy and timing of revascularisation in women with multivessel disease presenting with STEMI for PPCI.

## Conclusion

In STEMI patients with multivessel disease at PPCI, women under the age of 60 years had higher mortality, but in women older than 60 years comorbidity impacted outcome of revascularisation strategy in the long term.
